# Meningothelial Meningioma Component in a Prepubertal Testicular Teratoma

**DOI:** 10.1155/2020/6495321

**Published:** 2020-01-20

**Authors:** Ruchi Sinha, Madhu Kumari, Ranwir Kumar Sinha, Bindey Kumar

**Affiliations:** ^1^Department of Pathology, AIIMS, Patna, India; ^2^Department of Pathology, AIIMS, Patna, India; ^3^Department of Paediatric Surgery, AIIMS, Patna, India

## Abstract

Teratomas are nonseminomatous germ cell tumors composed of elements derived from more than one germinal layers (endoderm, mesoderm, and ectoderm). Malignant transformation of teratoma in the testis is well known; however, benign somatic neoplasm arising in a testicular teratoma is a rare occurrence. We report a case of meningothelial variant of meningioma arising in a pure and mature teratoma of the testis in a 5-year-old boy. Immunohistochemistry was positive for epithelial membrane antigen and vimentin. To our knowledge, this is the first report of meningothelial meningioma in a prepubertal testicular teratoma.

## 1. Introduction

Teratomas are tumors of germ cell origin with elements derived from more than one embryonic layer. They belong to the nonseminomatous germ cell tumor group. Teratomas can be classified as mature or immature depending upon the microscopic presence of well-differentiated tissue or immature fetal-like tissue, respectively [1]. The 2016 World Health Organization (WHO) classification differentiates testicular teratoma into prepubertal type and postpubertal type. It does not distinguish the postpubertal teratoma of the testis into mature and immature subtypes [2]. Here, we report a rare case of meningothelial variant of meningioma arising in a pure and mature testicular teratoma.

## 2. Case Report

A 5-year-old male patient presented with complaint of the left-sided testicular enlargement. On clinical examination, nontender testicular swelling was palpable. Ultrasonography showed a solid-cystic mass with multiple internal septations. On colour Doppler, the lesion appeared hypovascular. No extratesticular extension was observed. The right testis was of normal size and echotexture. Bilateral spermatic cords were normal. No metastatic foci or lymphadenopathy was detectable. Chest radiograph was normal. Serum testicular tumour markers were within normal range. A left orchiectomy was performed and sent for histopathology.

Grossly, the left orchiectomy specimen measured 3.4 × 1.5 × 1 cm. On cut section, a solid-cystic area measuring 1.4 × 0.5 cm was seen within the testis. Tissue was focally hard. The lesion was located entirely within the confines of the testis.

**Figure 1:**
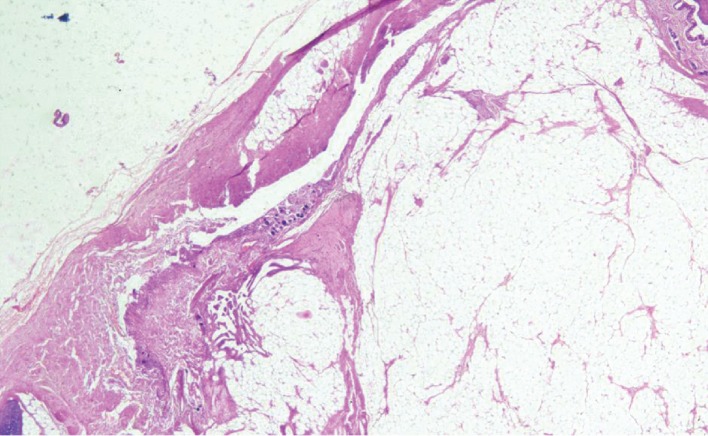
Tumour composed of mature tissues derived from different germinal layers (H&E; 4x).

**Figure 2:**
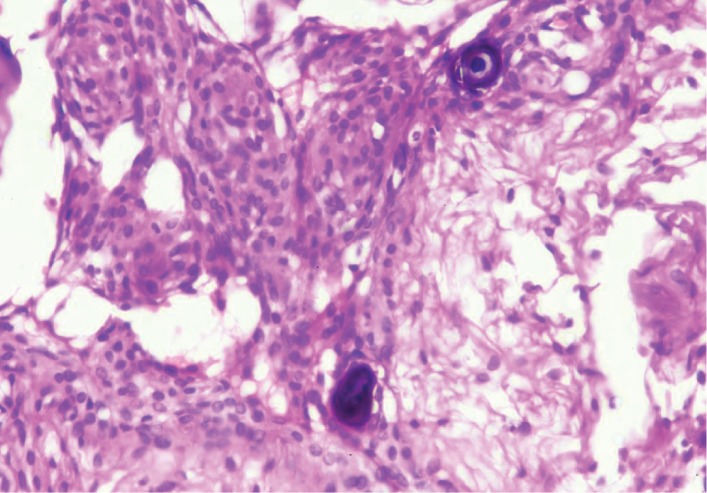
Whorled arrangement of meningothelial cells with the presence of psammoma bodies (H&E; 10x).

**Figure 3:**
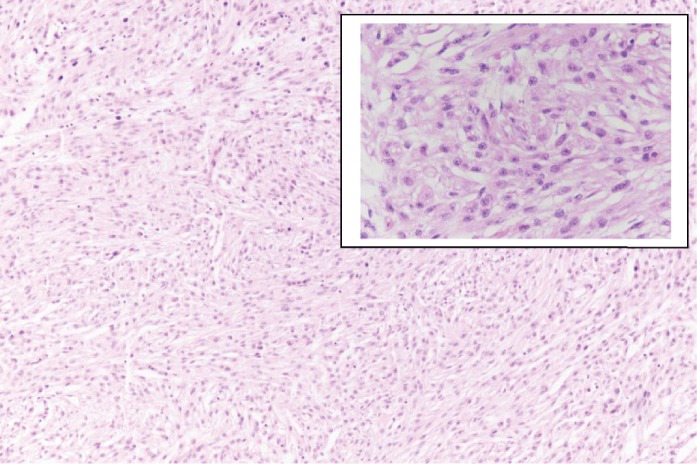
Meningothelial meningioma component with whorls of meningothelial cells (H&E; 10x. Inset: H&E; 40x).

On microscopic examination, multiple sections from the left testicular swelling showed a tumour composed of mature tissue derived from all the three germ layers—mature cartilage tissue, cystic spaces lined respiratory epithelium, glial tissue, adipose tissue, and muscle (Figure 1). In the solid area, admixed with the other teratomatous components, meningioma-like areas were identified which showed whorled arrangement of cells. These cells had oval nucleus, eosinophilic cytoplasm, and indistinct cell borders. Few psammoma bodies were also seen (Figures 2 and 3). This meningothelial component accounted for a major portion (more than 10%) of the entire teratomatous tissue and was present in all the five slides that contained teratoma. Morphologically, the pattern of growth resembled meningioma (whorled growth pattern) than meningothelial proliferation. No component of germ cell neoplasm in situ (GCNIS) was found. No microscopic evidence of extratesticular extension or lymphovascular invasion was found.

**Figure 4:**
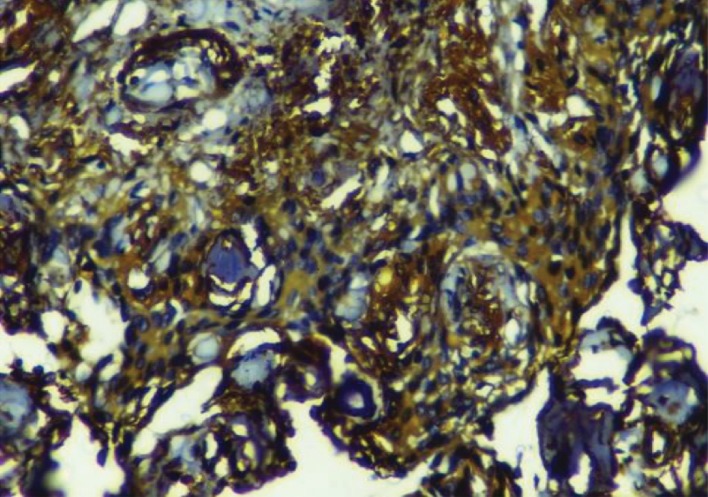
Meningothelial cells showing strong and diffuse positivity for vimentin (IHC; 40x).

**Figure 5:**
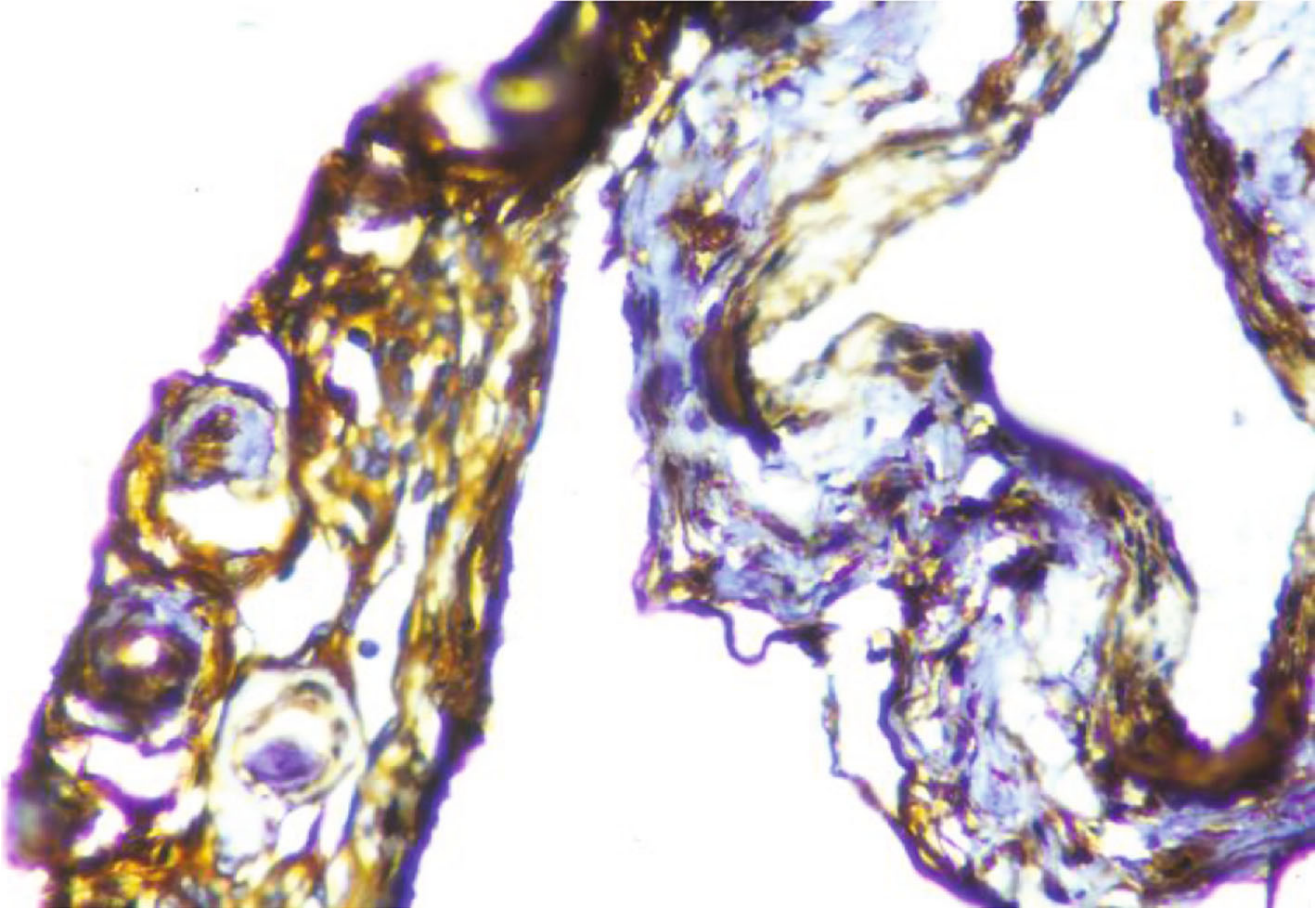
Positive staining for epithelial membrane antigen in meningothelial cells (IHC; 40x).

On immunohistochemistry (IHC), the meningiomatous component showed diffuse strong vimentin and epithelial membrane antigen (EMA) positivity (Figures 4 and 5).

## 3. Discussion

Teratomas are germ cell tumours (GCT). They can be gonadal or extragonadal in location. It is the second most common paediatric GCT of the testis [3]. They account for 5% to 10% of all testicular neoplasms [4]. It can present as an insidiously growing testicular mass or as a painful lesion if associated with hemorrhage [5].

The 2016 WHO classification differentiates testicular teratoma into prepubertal type and postpubertal type. It does not distinguish mature from immature teratoma of the postpubertal testis [2]. Prepubertal teratomas show organoid architecture with minimal cytological atypia [6], are not associated with GCNIS [7], usually lack 12p abnormality [8], and rarely metastasize [9].

Teratoma comprises of different types of tissues arising from two or more germinal layers. Thus, a variety of secondary somatic, both benign and malignant, neoplasms can arise from any of the three germ cell layers of a teratoma. Secondary somatic neoplasms are thought to arise due to overgrowth of a particular component of the teratoma [10]. Malignant neoplasms are more commonly encountered as compared to benign tumours. The most common somatic malignancy arising in teratoma of testis is sarcoma [11]. Identifying the biological nature and extent of spread of the secondary nongerm cell somatic-type neoplasm has prognostic significance [4, 12]. Patients in whom malignant component is localized to the testis having better prognosis than those in whom the malignant component metastasizes [13].

Reports of secondary benign somatic tumors arising in teratomas are very rare. They are more commonly encountered in the ovary as compared to the testis [4]. Secondary benign nongerm cell-type tumor in testicular teratoma is infrequent.

Ectodermal-derived benign tumours arising in testicular teratoma are extremely rare. On extensive search of English literature, only two cases of meningioma have been reported. One case was of a microcystic variant of meningioma which developed in mixed germ cell testicular teratoma in a 29-year-old man [4] and second case report is of a psammomatous variant arising in a pure testicular teratoma in a 46-year-old male [12].

Meningothelial proliferations containing psammoma bodies have been reported in mature testicular teratoma [14, 15]. Meningothelial proliferations are seen microscopically as interanastomosing slit-like channels lined by spindle or cuboidal cells within a dense fibrocollagenous stroma. These are mostly seen in peripheral parts of the teratoma and in close proximity of peripheral nerve bundles, glial tissue, and skin with abundant pilosebaceous unit. Scattered pigment cells and psammomatous concretions can be present. Whorled nests of meningothelial cells are rarely found in meningothelial proliferations [16]. To label such a proliferation as meningioma, it should constitute a major component of the teratoma, as in the present case (more than 10%). Allen et al. in their case report of microcystic meningioma had meningomatous component accounting for approxmitaley 15% of the teratomatous tissue [4]. Outgrowth of the meningothelial elements in the presence of psammoma bodies indicates the presence of benign meningioma within a teratoma [17].

Our case has two interesting features; it is not only the first case of meningioma arising in a pure prepubertal testicular teratoma in a 5-year-old boy but is also the first case of meningothelial variant. This is an extremely rare occurrence in a testicular teratoma and adds to the medical literature a unique case of benign tumor originating in a prepubertal testicular teratoma.
